# Comparison of first-line treatments for elderly patients with diffuse large B-cell lymphoma: A systematic review and network meta-analysis

**DOI:** 10.3389/fimmu.2022.1082293

**Published:** 2023-01-04

**Authors:** Yangyang Wang, Xiyang Ren, Keke Huang, Xue Liang, Lianfang Pu, Linhui Hu, Zhimin Zhai

**Affiliations:** Department of Hematology/Hematological Lab, The Second Hospital of Anhui Medical University, Hefei, Anhui, China

**Keywords:** diffuse large B-cell lymphoma, network meta-analysis, clinical decision-making, therapy, monoclonal antibody

## Abstract

**Background:**

The incidence of DLBCL in elderly patients has been gradually increased. Considering their comorbidities and performance status, the first-line standard treatment hasn’t been determined for the elderly.

**Methods:**

We performed a systemic review and network meta-analysis to compare the efficacy and safety of all eligible regimens as first line treatment for elderly patients with DLBCL. We searched PubMed, Cochrane Library, and Embase Library proceedings up to March 2022.

**Results:**

Our search yielded thirteen trials including 1839 patients. R2CHOP21 showed the best PFS with a statistical difference and the most favorable OS without a statistical difference. RCOMP showed the most clinical benefits in EFS, CR and OR with no significant difference. The point estimate was in favored improved DFS with RCHOP14 than RCHOP21, although this was not statistically significant. In a subgroup analysis concerning 3-4 grade AEs revealed R-COMP was associated with a decrease in grade III/IV neutropenia and cardiac toxic events; RminiCEOP was associated with the lower rates of 3-4 grade anemia, thrombocytopenia and infection; RCHOP21 had the lowest rate of 3-4 grade AE of neurotoxicity.

**Conclusion:**

The findings of our meta-analysis indicated that R2CHOP21 provided the best disease control in PFS and represented an optimal first-line treatment option in the elderly with DLBCL. Furthermore, RCOMP, RminiCEOP and RCHOP21 exhibited lower rates in different 3-4 grade AEs and might be reasonable treatment options in the elderly with poor general conditions.

## 1 Background

Diffuse large B-cell lymphoma (DLBCL) is very common in adult lymphoma, and the incidence has gradually increased in recent years (1). Most DLBCL cases occur in patients aged 60-70 years old at diagnosis, and approximately 40% are over 70 years old (2, 3). The advanced age among DLBCL patients at presentation demands that we place our focus on the management of DLBCL in elderly and very elderly patients (4).

It is well-known that rituximab + CHOP (cyclophosphamide, doxorubicin, vincristine, and prednisolone plus rituximab) is the fundamental and standard regimen for treatment of B-cell lymphoma, especially for DLBCL (5, 6). R-CHOP regimen has also been proven to be a suitable treatment for elderly patients with DLBCL, mainly in patients with low-risk (7). Elderly patients with high-risk cannot tolerate standard doses due to factors such as age, comorbidities, or performance status (8). Different protocol, such as dose-adjusted CHOP chemotherapy with rituximab (DA-POCH-R), was presented as the alternatively recommended treatment (9). Elderly patients can achieve a long-term remission rate of 50-60% after receiving attenuated R-CHOP therapy (10, 11). The trial (NCT01148446) aiming at compared standard R-CHOP with RminiCEOP (epirubicin, cyclophosphamide, vinblastine and prednisone with rituximab), and demonstrated that DLBCL patients with low-risk disease and over 72 years had a better outcome on R-miniCEOP compared to R-CHOP (12). Additionally, several studies analyzed the impact of the dose-dense regimen and treatment cycles. For instance, the trial RICOVER60 found that no significant differences were identified between 6 and 8 cycles of the R-CHOP14 regimen administered in DLBCL patients older than 60 years (13). As for the analysis of the dose-dense regimen, one trial was conducted by the GELA group and limited to elderly population; another trial was run by the British National Investigation (BNLI) among patients of all age (14, 15). The results of these two studies involving more than one thousand participants both failed to identify significant differences of benefits in the arm of dose-dense regimen. In addition to focusing on improving the survival outcomes, the adverse events of the therapies for the elderly are also important measuring factors. Previous studies reported that adverse events, involving cardiac and hematologic toxicity, were common in older patients during the R-CHOP regimen (13, 14). Substituting doxorubicin (gemcitabine or etoposide) with less cardiotoxic anthracyclines (e.g., epirubicin and pegylated doxorubicin) or other molecules might be a safe and effective approach. For DLBCL patients ≥60 years old, R-COMP (with Myocet ^®^ instead of conventional doxorubicin) was similar in efficacy to R-CHOP, but unfortunately no significant reduction in early cardio-toxicity was observed (16). The replacement of etoposide for doxorubicin in standard-dose R-CHOP (named as R-CEOP) was recommended to be used in patients who have a contraindication to anthracyclines; however, it is unclear whether this compromises clinical outcomes (17). These data strongly indicated that the first-line treatment options for the elderly need to be further explored and discussed.

To better define and tailoring the best frontline therapeutic strategies for elderly DLBCL patients among current and past available treatment approaches, we carried out this systematic review and network meta-analysis comprising all available randomized clinical trials to date.

## 2 Material and methods

### 2.1 Systematic review of the literature

The search strategy of our work comprised terms defining DLBCL and related disorders, elderly, and a sensitive filter strategy for randomized clinical trials. The search strategy for all the databases used is available in **Supplementary Table 1**.** **A bibliographic search of Pubmed, Cochorane and EMBASE databases for all accessible published randomized clinical trials (RCTs) was performed up to 20/3/2022. As for all retrieved RCTs and previous systematic reviews, we also hand-searched their references. Two authors (Linhui Hu and Yangyang Wang) conducted the literature search and study selection independently, with divergence reviewed and solved by consensus.

Inclusion criteria were strictly selected according to PICOS criteria, including population, intervention, comparator, outcome, and study design (18). The studies included in our analysis were randomized clinical head-to-head trials. The population analyzed involved newly diagnosed DLBCL patients who have not received treatment (over 60 years of age). Interventions included all regimens with R-CHOP, CHOP, R-CEOP, R-COMP, and any therapy applied in elderly DLBCL patients. Any comparator was brought in consideration. The primary efficacy outcomes were overall survival (OS), progression-free survival (PFS), event-free survival (EFS), and disease-free survival (DFS). Tumor responses were classified as complete response (CR) and overall response (OR), considering the criteria proposed by the International Workshop (19). Adverse events (AEs) included infection, hematological, cardiovascular, and neurological events. All the studies were published in English.

We excluded articles that only studied subgroups within the selected population (e.g., young patients); did not analyze efficacy outcomes mentioned above; did not present hazard ratio (HR) data; did not provide the data used for calculating the clinical outcome indicators we need, or were published in a language other than English.

### 2.2 Data extraction and quality assessment

The data extraction on the first author, treatment blinding, publication year, region, sample sizes, general characteristics of the population, therapeutic regimen and clinical outcomes were retrieved and summarized independently by two authors (Linhui Hu and Yangyang Wang) following Cochrane Collaboration guidelines. As for the identical study that published clinical outcomes based on different follow-up times, we extracted the most recent data in our analysis.

The bias risk of all the enrolled RCTs was assessed by two authors (Yangyang Wang and Xiyang Ren) using the Cochrane Risk of Bias Tool, involving seven items: random sequence generation, allocation sequence concealment, blinding of population and personnel, blinding of outcome evaluation, incomplete outcome data, selective outcome reporting, and other sources of bias (20). RCTs can be classified as low, high, or vague risk of bias.

### 2.3 Network meta-analysis

The hazard ratio (HR) for survival outcomes (OS, PFS, EFS, and DFS), the odds ratio for binary outcomes of treatment responses (CR, OR, and grade 3-4 AEs), and their 95% CIs were employed to measure clinical outcomes and safeties.

First, the network meta-analysis was performed by employing the mtc.model and mtc.run functions of the gemtc R package (21). These functions mentioned above implemented Bayesian methods that combine direct and indirect evidence. Fixed and random-effect models were considered and compared by deviance information criteria (DIC). The model which best adapts to the network (with the lowest score of DIC) was determined, constructing a value of 5 as the minimum relevant difference between the two alternatives (22). Second, a network of binary clinical outcomes including OR, CR, and AEs was established within all the enrolled studies and was used to specify the relationship among odds ratios across studies in order to compare different treatments in elderly DLBCL patients. Moreover, as for each outcome, the probability of every treatment was estimated at each possible rank, and the distribution of each regimen’s probabilities was presented in histograms.

## 3 Results

### 3.1 Systematic review and characteristics of all trials

The literature search in our study yielded 914 eligible articles. Thirteen studies fulfilled our inclusion criteria (**Supplementary Figure 1**) (6, 7, 12, 13, 16, 23–30). It contained 11 studies for OS, 10 studies for PFS, 4 studies for EFS, and 2 studies for DFS (The detailed characteristics of all the included trials are shown in **Table 1**). As for OR and CR, we analyzed 8 studies, and the details of these included studies were presented in **Supplementary Table 2**. Besides, 12 studies containing 7 AEs were evaluated in our study (**Supplementary Table 3**). The trials selected in our study were published between 2006 and 2022, among which 1839 patients were enrolled in the subsequent analyses. The sample sizes of all the involved studies ranged from 90 to 613. The follow-up time ranged from 34.5 to 92 mouths. The median age of the enrolled patients ranged from 65 to 74 years. These patients enrolled in our study received 12 different treatment options: 1) Cyclophosphamide, Doxorubicin, Vincristine, and Prednisone plus Rituximab every 14 days (RCHOP14), 2) Cyclophosphamide, Doxorubicin, Vincristine, and Prednisone plus Rituximab every 21 days (RCHOP21), 3) Cyclophosphamide, Doxorubicin, Vincristine, and Prednisone every 14 days (CHOP14), 4) Cyclophosphamide, Doxorubicin, Vincristine, and Prednisone every 21 days (CHOP21), 5) Cyclophosphamide, Pixantrone, Vincristine, and Prednisone plus Rituximab (RCPOP), 6) Cyclophosphamide, Epirubicin (50mg/m^2^), Vincristine, and Prednisone plus Rituximab (RminiCEOP) every 21 days, 7) Cyclophosphamide, Doxorubicin, Vincristine, and Prednisone plus Obinutuzumab (G-CHOP), 8) Cyclophosphamide, Epirubicin (70mg/m^2^), Vincristine, and Prednisone plus Rituximab (RCEOP), 9) Dose-adjusted (DA) Etoposide, Prednisone, Vincristine, Cyclophosphamide, and Doxorubicin plus Rituximab (DAEPOCHR), 10) Cyclophosphamide, Vincristine, Non-pegylated liposomal doxorubicin (Myocet^®^) and Prednisone plus Rituximab (RCOMP), 11) Cyclophosphamide, Doxorubicin, Vincristine, and Prednisone plus Rituximab every 21 days, additionally received 25 mg of lenalidomide daily days 1-10 of each cycle (R2CHOP21), 12) Cyclophosphamide, Doxorubicin, Vincristine, and Prednisone plus Rituximab every 14 days, additionally received 25 mg of lenalidomide daily days 1-10 of each cycle (RRCHOP14).

**Table 1:** Baseline Characteristics of Studies Included in the Network Meta-analysis of Patients With DLBCL.

First author	Year	Region	Sample size	Median age	Age range	Male sex	Median follow up (Months)	Stage	ECOG	IPI	Study enduration	Treatment A	Treatment B	Sample size A	Sample size B	HR	LCI	HCI	Outcome
Pfreundschuh (13)	2008	European	613	——	61-80	338(55)	34.5	I-IV	0-4	1-5	2000.07.01-2005.06.14	CHOP14	RCHOP14	307	306	0.51	0.4	0.65	EFS
																0.50	0.38	0.67	PFS
																0.63	0.46	0.85	OS
Habermann (7)	2006	Canada	178	——	——	——	42	——	——	——	1998.2-2001.7	RCHOP21	CHOP21	267	279	0.72	0.52	1	OS
Coiffier (6)	2010	France	399	70	60-80	——	120	II-IV	——	——	——	CHOP21	RCHOP21	197	202	1.59	1.26	2.01	PFS
																1.63	1.27	2.08	OS
																1.80	1.34	2.42	DFS
Nowakowski (25)	2021	many countries	201	——	>=60	——	27.1	II-IV	0-2	2-5	2013.08-2017.01	R2CHOP21	RCHOP21	105	96	0.74	0.429	1.276	OS
																0.73	0.456	1.169	PFS
Vitolo (23)	2017	Italy	568	——	65-86	——	29	——	——	——	2011.07-2014.06	G-CHOP	RCHOP21	288	280	0.99	0.73	1.34	PFS
Merli (12)	2012	Italy	224	72	65-86	104(46)	42	III-IV	0-3	0-3	2003.01-2006.12	RCHOP21	RminiCEOP	110	114	1.09	0.71	1.67	OS
																0.89	0.62	1.28	EFS
Sancho (16)	2020	Spain	90	74	60-86	41(46)	42	I-IV	0-4	0-5	2013.10-2016-02	RCOMP	RCHOP21	45	45	0.58	0.31	1.07	EFS
																0.80	0.41	1.55	PFS
																0.88	0.39	1.98	OS
Xu (30)	2019	China	243	65	63-70	139(57)	45.7	I-IV	0-2	low-high	2013.05.15-2016.3.16	RCEOP	RCHOP21	121	122	1.09	0.72	1.66	PFS
																1.02	0.63	1.64	OS
Delarue (27)	2013	France	602	70	60-80	334(55)	56	I-IV	0-4	0-5	2003.12-2008.11	RCHOP14	RCHOP21	304	298	1.04	0.82	1.31	EFS
																1.111	0.831	1.485	PFS
																0.80	0.58	1.10	DFS
																1.038	0.74	1.455	OS
Bartlett (26)	2019	America	208	——	60-86	——	62.4	III-IV	0-2	0-5	2005-2013	DAEPOCHR	RCHOP21	102	106	1.15	0.74	1.77	PFS
																1.17	0.72	1.91	OS
Ku¨ hnl (29)	2017	UK	604	67	60-88	306(51)	77.7	I-IV	0-2	1-5	——	RCHOP21	RCHOP14	301	303	1	0.78	1.29	PFS
																0.95	0.73	1.25	OS
Lugtenburg (24)	2020	European	285	——	66-80	——	92	II-IV	0-4	low-high	2007.11.14-2012.04.06	RCHOP14	RRCHOP14	146	139	1.20	0.93	1.54	PFS
																1.25	0.95	1.65	OS
																1.25	0.98	1.61	FFS
Herbrecht (28)	2012	France	124	68	65	63(51)	——	——	0-2	0-5	2005.11.28-2008.1.31	RCPOP	RCHOP21	38	37	2.65	1.09	6.46	OS

### 3.2 Risk of bias in the included studies

**Supplementary Table 4** displayed the risk of bias judgments for all the included trials. To sum up, the majority of trials used randomized under-reporting and concealment techniques, and participants and staff were not blinded. These factors impaired the risk assessment.

### 3.3 Survival analyses

**Figure 1:**
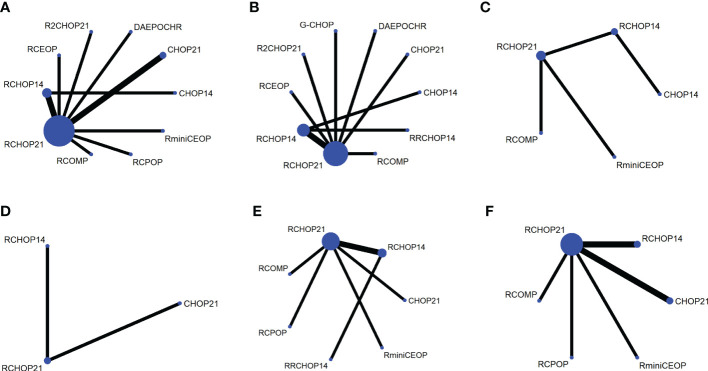
Network plot for each of the different outcomes assessed **(A)** OS, **(B)** PFS, **(C)** EFS, **(D)** DFS, **(E)** CR, **(F)** OR.

The network was performed to implement numerous drug comparisons when added to traditional therapy or dose-adjustments based on conventional regimens (**Figure 1**). For nearly two decades, the first-line treatment in patients with DLBCL has been RCHOP21. Based on this factor, we used the classic RCHOP21 regimen as a standard when exploring the best treatment options for elderly DLBCL patients and compared the pros and cons among these regimens.

**Figure 2:**
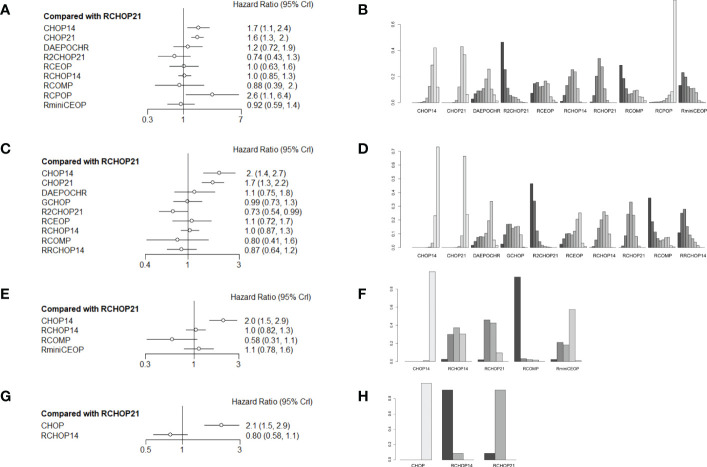
**(A)** Forest plot of overall survival; **(B)** Rank probability of overall survival; **(C)** Forest plot of progression-free survival; **(D)** Rank probability of progression-free survival; **(E)** Forest plot of event-free survival; **(F)** Rank probability of event-free survival; **(G)** Forest plot of disease-free survival; **(H)** Rank probability of disease-free survival.

More than 1000 patients were analyzed for the OS analysis, which included 9 treatment arms (**Figure 1A**). Notably, R2CHOP21 was superior to RCHOP21 in improving OS, but there existed no significant difference existed between R2CHOP21 and RCHOP21 (HR 0.74 [95%CI, 0.43-1.3], **Figure 2A**). Similarly, RCOMP and RminiCEOP produced better OS in elderly DLBCL patients, although no statistical difference was determined (RminiCEOP, HR 0.92 [95%CI, 0.59-.1.4]; R-COMP, HR 0.88 [95%CI, 0.39-2.0]; **Figure 2A**). A subgroup analysis for OS of both RCEOP and RCHOP14 showed similar point estimates compared to RCHOP21 with no difference (**Figure 2A**). DAEPOCHR, CHOP14, CHOP21 and RCPOP were inferior in OS improvement than RCHOP21, and the statistical difference between the latter three schemes was very obvious (DAEPOCHR, HR 1.2 [95%CI, 0.72-1.9]; CHOP14, HR 1.7 [95%CI, 1.1-2.4]; CHOP21, HR 1.6 [95%CI, 01.3-2]; RCPOP, HR 2.6 [95%CI, 1.1-6.4]; **Figure 2A**). In a word, R2CHOP21 was recommended as the first choice for improving OS of elderly DLBCL treatment based on **Figure 2B**.

In terms of PFS, 9 treatment arms were analyzed, and 1022 patients were involved (**Figure 1B**). R2CHOP21 showed the best PFS than RCHOP21 with a statistically significant difference (HR 0.73 [95%CI, 0.54-0.99], **Figure 2C**). We obtained more PFS benefits from RCOMP and RRCHOP14 than from RCHOP21, while there was no statistically significant difference (RCOMP, HR 0.80 [95%CI, 0.41-1.6]; RRCHOP14, HR 0.87 [95%CI, 0.64-1.2]; **Figure 2C**). GCHOP showed slight advantage in PFS over RCHOP21 with no obviously difference (**Figure 2C**). No difference was found between RCHOP14 and RCHOP21 in improving the PFS of elderly patients (**Figure 2C**). Besides, DAEPOCHR and RCEOP were all inferior to RCHOP21, although no difference existed (**Figure 2C**). No benefits of PFS were identified among CHOP14 and CHOP21 compared with RCHOP21 (CHOP14, HR 2.0 [95%CI, 1.4-2.7]; CHOP21, HR 1.7 [95%CI, 1.3-2.2]; **Figure 2C**). Eventually, R2CHOP21 was recommended as the first option in all the simulations (**Figure 2D**).

As for EFS, there were 4 treatment arms, including 613 patients (**Figure 1C**). Our results suggested that the EFS benefit was more related to RCOMP than RCHOP21, although there was no statistically significance (HR 0.58 [95%CI, 0.31-1.1], **Figure 2E**). No sign of difference was identified between RCHOP14 and RCHOP21 (**Figure 2E**). CHOP14 and RminiCEOP were less effective in improving EFS compared with RCHOP21 (CHOP14, HR 2.0 [95%CI, 1.5-2.9]; RminiCEOP, HR 1.1 [95%CI, 0.78-1.6]; **Figure 2E**). Thus, RCOMP might be a reasonable treatment option based on the clinical benefits of EFS (**Figure 2F**).

Regarding DFS, the final indication of survival outcomes was assessed among 2 treatment arms involving 1001 patients. An advantage of DFS was determined in RCHOP14 than RCHOP21 without significant difference (HR 0.80 [95%CI, 0.58-1.1], **Figure 2G**). Adding the rituximab to the CHOP regimen exhibited a significant benefit to DFS than CHOP (HR 2.1 [95%CI, 1.5-2.9], **Figure 2G**). Therefore, considering the improvement of DFS, RCHOP14 was thought to be an appropriate treatment option (**Figure 2H**).

**Figure 3:**
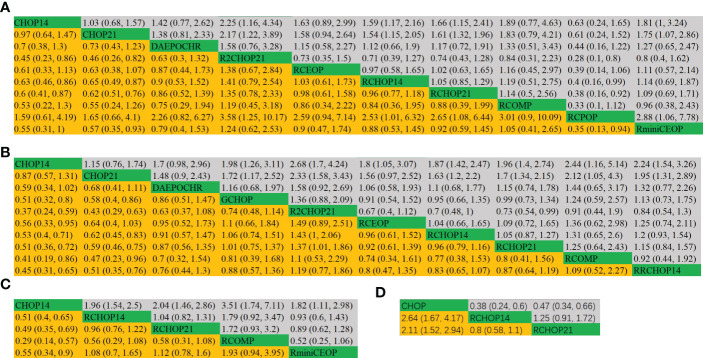
League table of **(A)** OS, **(B)** PFS, **(C)** EFS and **(D)** DFS.

The league tables concerning these four mainly clinical outcomes are shown in **Figure 3**. Estimate values were evaluated by the mean differences with 95% confidence intervals (CIs) in parentheses. R2CHOP21, RCHOP14 and RCHOP21 were found to be superior in improving the OS of DLBCL patients (**Figure 3A**). Then for PFS, R2CHOP21 were shown to be superior to any other regimen (**Figure 3B**). It is worth noting that the R2CHOP21 regimen brings out the most optimal results, whether in improving OS or PFS. As for EFS, the mean differences were small or very uncertain. (**Figure 3C**). As oppose to the previous conclusions, CHOP was superior to either RCHOP14 or RCHOP21 in improving DFS (**Figure 3D**).

### 3.4 Analyses of complete remission and overall response rate

**Figure 4:**
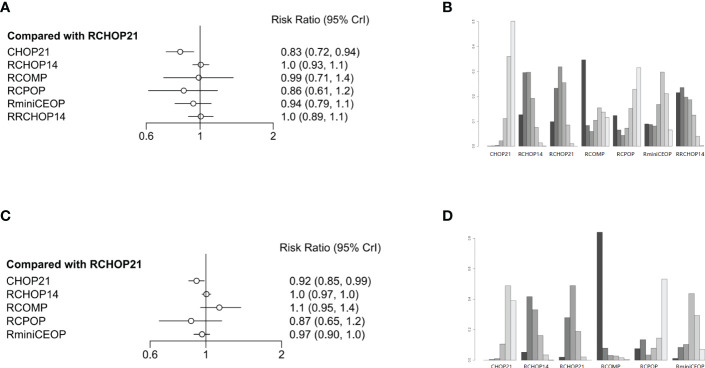
**(A)** Forest plot of complete response; **(B)** Rank probability of complete response; **(C)** Forest plot of overall response; **(D)** Rank probability of overall response.

The network designed for evaluating the CR and OR in simultaneous comparisons of different regimens is presented in **Figure 4**.

Concerning CR, four treatment arms including 1861 patients were brought in the analysis (**Figure 1E**). Both RRCHOP14 and RCHOP14 regimens showed similar rates of overall responses compared with RCHOP21 (RRCHOP14, HR 1.0 [95%CI, 0.89-1.1]; RCHOP14, HR 1.0 [95%CI, 0.93-1.1]; **Figure 4A**). Additionally, we didn’t obtain any sign of OR improvement in RCOMP, RCPOP and RminiCEOP compared to RCHOP21 (RCOMP, HR 0.99 [95%CI, 0.71-1.4]; RCPOP, HR 0.86 [95%CI, 0.61-1.2]; RminiCEOP, HR 0.94 [95%CI, 0.79-1.1]; **Figure 4A**). Trends on CHOP21 presented exhibited inferior effects on OR over RCHOP21 (CHOP21, HR 0.83 [95%CI, 0.72-0.94], **Figure 4A**). According to rankogram, RCMOP might be the better choice for CR (**Figure 4B**).

Regarding OR, there were five treatment arms with 2407 patients (**Figure 1F**). RCOMP exhibited more clinical benefits in OR than RCHOP21 with no statistically significant difference (RCOMP HR 1.1 [95%CI, 0.95-1.4], **Figure 4C**). RCHOP14 exhibited similar effects on improving OR in DLBCL patients compared with RCHOP21 (RCHOP14 HR 1.0 [95%CI, 0.97-1.0], **Figure 4C**). CHOP21, RCPOP, and RminiCEOP were all inferior to RCHOP21 in the improvement of OR (CHOP21 HR 0.92 [95%CI, 0.85-0.99]; RCPOP, HR 0.87 [95%CI, 0.65-1.2]; RminiCEOP, HR 0.97 [95%CI, 0.90-1.0]; **Figure 4C**). Synthetically, RCOMP was considered as the best choice for OR (**Figure 4D**).

**Figure 5:**
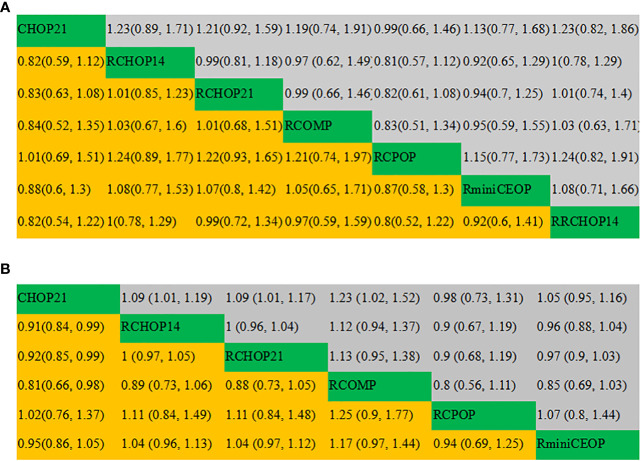
league table of **(A)** complete response, **(B)** overall response.

The league tables concerning these four clinical responses are shown in **Figure 5**.

### 3.5 Safety/toxicity analysis

**Figure 6:**
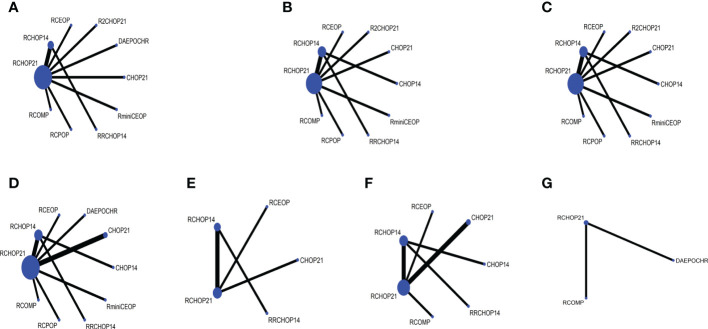
Network plot of **(A)** 3 to 4 grade neutropenia, **(B)** 3 to 4 grade anemia, **(C)** 3 to 4 grade thrombocytopenia, **(D)** 3 to 4 grade infection, **(E)** 3 to 4 grade neurotoxicity, **(F)** 3 to 4 grade cardiac toxic, **(G)** 3 to 4 grade neuropathy.

The risk of 7 different grade 3 to 4 AE groups were evaluated in our study: 3 to 4 grade neutropenia, 3 to 4 grade anemia, 3 to 4 grade thrombocytopenia, 3 to 4 grade infection, 3 to 4 grade cardiac toxic, 3 to 4 grade neurotoxicity, and 3 to 4 grade neuropathy. The network plot for each of the multiple AEs enrolled in our analysis was shown in **Figures 6A-G**. It is important to note that in AE groups, neutropenia was the most prevalent AE (**Supplementary Table 3**).

**Figure 7:**
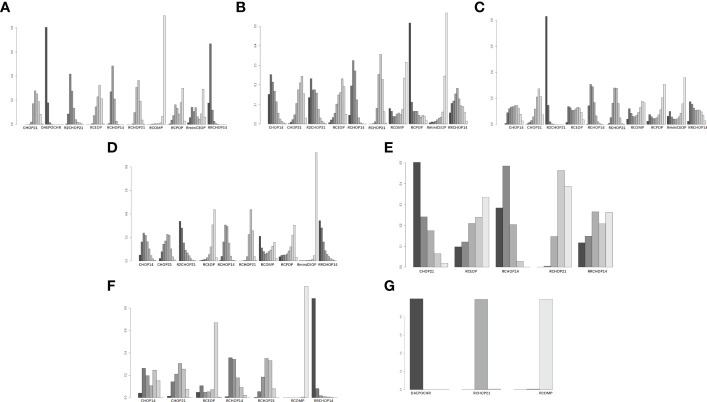
Rank probability of **(A)** 3 to 4 grade neutropenia, **(B)** 3 to 4 grade anemia, **(C)** 3 to 4 grade thrombocytopenia, **(D)** 3 to 4 grade infection, **(E)** 3 to 4 grade neurotoxicity, **(F)** 3 to 4 grade cardiac toxic, **(G)** 3 to 4 grade neuropathy.

In consideration of 3-4 grade neutropenia, neuropathy and cardiac toxic events, Myocet^®^ was a best substitution of doxorubicin in RCHOP on account of the lowest incidence of the three AEs (**Figures 7A, F**). Additionally, RminiCEOP was related to the lower rates of anemia and thrombocytopenia AEs (**Figures 7B, C**), and this regimen was also the first option for DLBCL patients with 3-4 grade infection (**Figure 7D**). Based on 3 to 4 grade neurotoxicity analysis, RCHOP21 may offer better advantages in preventing the therapy toxicity in elderly patients with DLBCL (**Figure 7E**).

## 4 Discussion

The most prevalent NHL histotype, DLBCL, peaked in incidence in the sixth decade (31). The standard regimen for DLBCL patients is Rituximab-CHOP; however, in elderly patients, there is currently no known standard first-line immuno-chemotherapy regimen. Our study, which is the first to thoroughly analyze the efficacy of various treatment regimens based on their clinical advantages and safety profiles, included 13 head-to-head phase 2 and 3 RCTs with 1839 DLBCL patients. Pairwise comparisons and ranking of various treatment options were evaluated as part of the analysis, which was carried out in a Bayesian hierarchical modeling framework using both direct and indirect evidence. When improved clinical outcomes and fewer toxicities are required, the findings of our network-analysis did in fact offer clinicians crucial guidance in order to choose from the first-line regimens that are already on the market.

Our NMA suggested that the adjustment to front-line R-CHOP21 regimen of DLBCL led to some improvement in clinical outcomes and treatment responses in elderly patients, although we only observed improved disease controls with statistically significance in R2CHOP21 compared to RCHOP21. A previous NMA by Pasvolsky et al. reached a similar conclusion, but their study was restricted to analyzing the response to R-CHOP in combination with another drug (R-CHOP + X) for DLBCL patients of all ages (32). The treatment options included in our study were more diverse compared to the NMA by Pasvolsky et al., and our study highlighted the treatment measurement in elderly patients. Undoubtedly, personalized clinical decision-making is a complex process for any individual patient. Prolonged survival time, increased response rates, reduced side effects, patient comorbidities, and patient preferences should be taken into account. In light of this, the objective of this study was to provide clinicians with evidence of efficacy from large randomized controlled trials rather than a design for identifying a regimen that should be most effective for all elderly patients. Clinicians may be better weigh the balance between potential advantages and risks when selecting the optimal treatment choice for any individual patient with the help of pairwise efficacy comparisons and ranking of the various treatment alternatives in this network meta-analysis.

Undeniably, in the current era of frequent emergence of new drugs, rituximab, as the first tumor-targeted drug and the first monoclonal antibody in history, is of epoch-making significance for the treatment of lymphoma, particularly DLBCL (33). The birth of rituximab has improved the 5-year OS of patients with aggressive B-cell lymphoma represented by DLBCL by at least 15%, and the cure rate has been significantly improved (6). This phenomenon has resulted in a huge improvement in efficacy and survival prognosis for B-cell lymphoma around the world. In view of the greater clinical benefits that rituximab brings to DLBCL patients, many researchers have begun to wonder whether RCHOP can be used in combination with other immuno-therapeutic drugs to further improve the clinical benefits of DLBCL patients. Lenalidomide, a new-generation immuno-modulator, may have potential effects on other mechanisms, such as immunomodulation and enhancement of antibody-dependent cytotoxicity in DLBCL patients (34, 35). Because of its novel activated mechanism, synergy with rituximab or chemotherapy, and moderate toxicity profile, lenalidomide has long been a key candidate for chemo-immunotherapy in the frontline treatment of DLBCL, and it was allowed to be safely combined with R-CHOP. Lugtenburg et al. investigated whether R-CHOP with early rituximab intensification (R2CHOP) could improve the clinical outcomes of patients, but it failed to have any effect on this intensification regimen (24). On the contrary, Nowakowski et al. proved that R2CHOP had an association with an obvious clinical benefit for PFS in most groups in 2021 after the results of the study by Lugtenburg et al. were published (24, 25). Our NMA revealed that R2CHOP21 created improved benefits in OS with no statistical significance compared with RCHOP21, but for PFS, this regimen was superior to RCHOP21 with statistical significance; meanwhile, other regimens did not exhibit any improvement in OS or PFS of elderly DLBCL patients. In terms of AEs assessment, only one study by Nowakowski et al. was included in our analysis; compared with the R-CHOP arm, the most common grade 3/4 adverse reactions of the R2CHOP21 arm were increased hematological AEs while all these toxicities did not bring about an increase in rates of therapy associated deaths or bleeding complications (25). This phenomenon implied that the adverse reactions in the R2-CHOP group were tolerated to some degree. In the future, it is still necessary to explore the mechanism of lenalidomide and the molecular biological characteristics of DLBCL in order to achieve more precise treatment.

Considering the main clinical outcomes, the place of R-CHOP21 in treatment options for total DLBCL patients remains important, while there exist some patients with poor efficacy on this regimen, notably in freshly treated DLBCL patients who are susceptible to refractory or early recurrence and have high-risk or other systemic problems (36). Due to the cardiotoxicity caused by doxorubicin, the implement of RCHOP should be restricted in elderly individuals. The drug’s interaction with the ferricion, which results in the generation of free radicals that contribute to lipid peroxidation and progressive myocyte destruction, causes doxorubicin-induced cardiotoxicity (37). Several schemes have been proposed to reduce cardiotoxicity caused by anthracyclines in elderly DLBCL patients, involving the implementation of slow infusions or decreased doses of doxorubicin, application of cardio-protective agents, substitution by other anti-neoplastic drugs, or selection of other anthracyclines with less cardiotoxic, such as epirubicin, liposomal formulations of doxorubicin, etc (38–40). Myocet ^®^, a non-polyethylene glycol liposome doxorubicin, has proven that its cardio-toxicity is low and its anti-tumor activity is analogous to that of conventional doxorubicin based on a clinical trial about breast cancer (37). Myocet^®^ is also linked to decreased mucositis and myelosuppression due to its pharmacokinetics and pharmacodynamic properties (41). In our study performed for elderly DLBCL patients, the use of non-pegylated doxorubicin instead of conventional doxorubicin (R-COMP) exhibited the best clinical benefits in EFS, CR, and OR, although no obvious significant difference was determined between RCOMP and RCHOP21. Similar to a previous study, Sancho et al. pointed out that the efficacy of the R-COMP group was analogous to R-CHOP (16). Moreover, our results demonstrated that RCOMP was associated with less 3-4 grade neutropenia, neuropathy, and cardiac toxic events. In a word, R-COMP is a feasible immuno-chemotherapy schedule for elderly DLBCL patients when RCHOP is not suitable for application. Epirubicin, another epi-isomer of doxorubicin with less cardiotoxicity has been a potential candidate for anthracycline dose intensification (38). Based on our analysis, RminiCEOP was related to less anemia and thrombocytopenia, and infection of grade III/IV. RminiCEOP could serve as an alternative therapy for elderly populations with hematological system complications.

In addition, our analysis assessed the possible superiority of the dose-dense regimen (R-CHOP14) compared with the standard regimen (R-CHOP21), but failed to identify a significant benefit for R-CHOP14 in elderly patients with DLBCL. Meanwhile, our results are consistent with previous work by Delarue et al. (27). Other protocols, such as DA-EPOCH-R, GCHOP, RCPOP, and CHOP have failed to demonstrate improved efficacy in elderly DLBCL patients compared to RCHOP21. It was obvious that RCPOP was inferior to RCHOP21 in OS, and either CHOP14 or CHOP21 was significantly less effective than RCHOP21 in almost all evaluations of clinical outcomes.

Nervelessness, the following issues should be considered carefully. Firstly, the patients included in our study have used granulocyte stimulating factor according to their conditions during the treatment process, so the treatment rankogram in analysis of 3 to 4 grade neutropenia in our results should be treated with caution. Secondly, there are some differences in the age of the population, follow-up time, treatment time arrangement, and so on. These differences might lead to some heterogeneity in our results. Thirdly, in our included studies, the Eastern Cooperative Oncology Group (ECOG) scores of patients ranged from 0 to 4, but mainly focus on 0 to 2, so that our conclusions were more suitable for patients with better performance status (ECOG ranged from 0 to 2), and may be usefulness in patients who with a poor performance status (ECOG ranged from 3 to 4). Further studies should be designed to solve this issues.

In general, our meta analysis only provided a reference value. Owing to the lack of direct comparisons of certain regimens or the insufficiencies of number and quality in the original studies, we made some indirect comparisons in this study based on the transitivity assumption. Further refinement of regimen comparisons lacking direct evidence is what we need to focus on.

## 5 Conclusion

Our network meta-analysis compares the widest range of treatment options to date, including R-CHOP, CHOP, R-COMP, R-CPOP, and so on. In general, our findings indicated that R2CHOP21 exhibited better improvement than RCHOP21 in different clinical outcomes. However, we only observed a statistically significant improvement in PFS; this phenomenon suggested that rituximab-based standard therapy upon CHOP has irreplaceable advantages as first-line therapy. In terms of decreasing toxicities from treatments, RCOMP, RminiCEOP and RCHOP21 exhibited lower toxicity in different AEs. Therefore, these findings could provide valuable recommendations for clinicians in making better decisions from multiple promising treatment options for elderly DLBCL patients by fully considering their clinical benefits and toxicity profiles.

## Data availability statement

The original contributions presented in the study are included in the article/**Supplementary Material**. Further inquiries can be directed to the corresponding authors.

## Author contributions

The literature search and study selection were conducted independently by two authors (YW and XR), with discrepancies reviewed and resolved by consensus (LH and ZZ). The data extraction was retrieved and summarized independently by two authors (YW and XR) following Cochrane Collaboration guidelines. The bias risk of all the enrolled RCTs was assessed by two authors (XL and LP) using the Cochrane Risk of Bias Tool. The data analysis were performed by three authors (YW, XR and KH). All authors contributed to the article and approved the submitted version.

## Supplementary material

Supplementary Figure 1 Systematic literature process of the search.
