# Supplementary material for: Comparison of first-line treatments for elderly patients with diffuse large B-cell lymphoma: A systematic review and network meta-analysis
Source: Front Immunol. 2023 Jan 4;13:1082293. doi: 10.3389/fimmu.2022.1082293 (PMC9845876; doi:10.3389/fimmu.2022.1082293)
Supplement: Supplementary file 1 [file Table_1.docx]

**Supplementary table 1.** Search strategy.

**Pubmed**

| aged | #1 | (((((((aged[MeSH Terms])) OR (elderly[Title/Abstract])) OR (older[Title/Abstract])) OR (old[Title/Abstract])) OR (aged, 80 and over[MeSH Terms]))) |
| --- | --- | --- |
| DLBCL | #2 | (((((((Lymphoma, Large B-Cell, Diffuse[MeSH Terms])) OR (Lymphoma, Large Lymphoid, Diffuse[Title/Abstract])) OR (Diffuse Large B Cell Lymphoma[Title/Abstract])) OR (Diffuse, Large B-Cell, Lymphoma[Title/Abstract])) OR (DLBCL[Title/Abstract]))) |
| RCT | #3 | ((randomized controlled trial[Publication Type] OR randomized[Title/Abstract] OR placebo[Title/Abstract])) |
| Search strategy | #1 AND #2 AND #3 | |

**Cochorane**

| DLBCL | #1 | (‘MeSH descriptor: [Lymphoma, Large B-Cell, Diffuse] explode all trees’)  OR (‘diffuse large B cell lymphoma OR DLBCL’) |
| --- | --- | --- |
| aged | #2 | MeSH descriptor: [Aged] explode all trees OR elderly OR older |
| Search strategy | #1 AND #2 | |

**Embase**

| DLBCL | #1 | ('diffuse large b cell lymphoma'/exp) OR ('dlbcl') OR (‘diffuse large b cell lymphoma’) |
| --- | --- | --- |
| aged | #2 | ('aged'/exp) OR ('elderly') OR (‘older’) |
| RCT | #3 | ('randomized controlled trial'/exp) OR ('rct') |
| Search strategy | #1 AND #2 AND #3 | |

**Supplementary table 2.** CR and OR of studies included in the network meta-analysis of patients with DLBCL (CR: complete response; OR: overall response; DLBCL: Diffuse large B-cell lymphoma).

| Study | t1 | t2 | Total  ARM A | Total  ARM B | CR | | OR | |
| --- | --- | --- | --- | --- | --- | --- | --- | --- |
|  |  |  |  |  | ARM A | ARM B | ARM A | ARM B |
| Coffier | RCHOP21 | CHOP21 | 202 | 197 | 154 | 124 | 168 | 136 |
| Habermann | RCHOP21 | CHOP21 | 267 | 279 | —— | —— | 206 | 212 |
| Merli | RCHOP21 | RminiCEOP | 110 | 114 | 80 | 78 | 105 | 105 |
| Delarue | RCHOP14 | RCHOP21 | 304 | 296 | 216 | 220 | 268 | 257 |
| Ku¨ hnl | RCHOP14 | RCHOP21 | 274 | 274 | 184 | 169 | 248 | 249 |
| Lugtenburg | RCHOP14 | RRCHOP14 | 146 | 139 | 128 | 122 | —— | —— |
| Sancho | RCHOP21 | RCOMP | 45 | 45 | 28 | 28 | 36 | 41 |
| Herbrecht | RCPOP | RCHOP21 | 61 | 63 | 31 | 37 | 35 | 41 |

**Supplementary table 3.** III/IV grade AEs of studies included in the network meta-analysis of patients with DLBCL (AE: adverse event; DLBCL: Diffuse large B-cell lymphoma).

| First author | t1 | t2 | Total  ARM A | Total  ARM B | Infection | | Neutropenia | | Anemia | | Thrombocytopenia | | Cardiac toxicity | | Neurotoxicity | | Neuropathy | |
| --- | --- | --- | --- | --- | --- | --- | --- | --- | --- | --- | --- | --- | --- | --- | --- | --- | --- | --- |
|  |  |  |  |  | Arm A | Arm B | Arm A | Arm B | Arm A | Arm B | Arm A | Arm B | Arm A | Arm B | Arm A | Arm B | Arm A | Arm B |
| Coffier | RCHOP21 | CHOP21 | 202 | 197 | 24 | 39 | —— | —— | —— | —— | —— | —— | 16 | 16 | 10 | 18 | —— | —— |
| Habermann | RCHOP21 | CHOP21 | 267 | 279 | 45 | 45 | 208 | 218 | 45 | 45 | 37 | 28 | 24 | 25 | —— | —— | —— | —— |
| Merli | RCHOP21 | RminiCEOP | 110 | 114 | 7 | 2 | 20 | 22 | 8 | 5 | 2 | 2 | —— | —— | —— | —— | —— | —— |
| Delarue | RCHOP14 | RCHOP21 | 304 | 295 | 50 | 51 | 224 | 189 | 68 | 51 | 48 | 58 | 4 | 11 | 16 | 16 | —— | —— |
| Ku¨ hnl | RCHOP21 | RCHOP14 | 301 | 203 | 71 | 71 | 23 | 36 | 6 | 14 | 22 | 37 | 2 | 9 | 23 | 36 | —— | —— |
| Xu | RCHOP21 | RCEOP | 122 | 121 | 30 | 23 | 108 | 104 | 25 | 23 | 12 | 11 | 2 | 0 | 8 | 8 | —— | —— |
| Lugtenburg | RCHOP14 | RRCHOP14 | 145 | 139 | 27 | 34 | 47 | 62 | 25 | 21 | 13 | 12 | 2 | 9 | 11 | 8 | —— | —— |
| Sancho | RCHOP21 | RCOMP | 45 | 45 | 5 | 7 | 22 | 13 | 3 | 3 | 4 | 4 | 7 | 0 | —— | —— | 1 | 0 |
| Nowakowski | R2CHOP21 | RCHOP21 | 121 | 123 | —— | —— | 68 | 56 | 36 | 25 | 41 | 16 | —— | —— | —— | —— | —— | —— |
| Herbrecht | RCPOP | RCHOP21 | 59 | 63 | 10 | 11 | 36 | 39 | 7 | 3 | 3 | 4 | —— | —— | —— | —— | —— | —— |
| Pfreundschuh | CHOP14 | RCHOP14 | 307 | 306 | 83 | 79 | —— | —— | 46 | 45 | 18 | 23 | 5 | 7 | —— | —— | 20 | 30 |
| Bartlett | DAEPOCHR | RCHOP21 | 237 | 243 | 40 | 26 | 83 | 43 | —— | —— | —— | —— | —— | —— | —— | —— | 44 | 8 |

**Supplementary table 4.** Risk of bias in the included studies.

| Study | Random sequence generation | Allocation  concealment | Blinding of participants  and personnel | Incomplete  outcome data | Selective reporting | Other bias |
| --- | --- | --- | --- | --- | --- | --- |
| Habermann 2006 | low | unclear | unclear | low | low | low |
| Pfreundschuh 2008 | low | unclear | unclear | low | low | low |
| Herbrecht 2012 | low | unclear | unclear | low | low | low |
| Merli 2012 | low | unclear | unclear | low | low | low |
| Delarue 2013 | low | unclear | unclear | low | low | low |
| Coiffier 2010 | low | unclear | unclear | low | low | low |
| Ku¨ hnl 2017 | low | unclear | unclear | low | low | low |
| Vitolo 2017 | low | unclear | unclear | low | low | low |
| Xu 2019 | low | unclear | unclear | low | low | low |
| Bartlett 2019 | low | unclear | unclear | low | low | low |
| Lugtenburg 2020 | low | unclear | unclear | low | low | low |
| Sancho 2020 | low | unclear | unclear | low | low | low |
| Nowakowski 2021 | low | unclear | unclear | low | low | low |
| Lugtenburg 2020 | low | unclear | unclear | low | low | low |
