# Supplementary figures and images for: Comparison of first-line treatments for elderly patients with diffuse large B-cell lymphoma: A systematic review and network meta-analysis
Source: Front Immunol. 2023 Jan 4;13:1082293. doi: 10.3389/fimmu.2022.1082293 (PMC9845876; doi:10.3389/fimmu.2022.1082293)

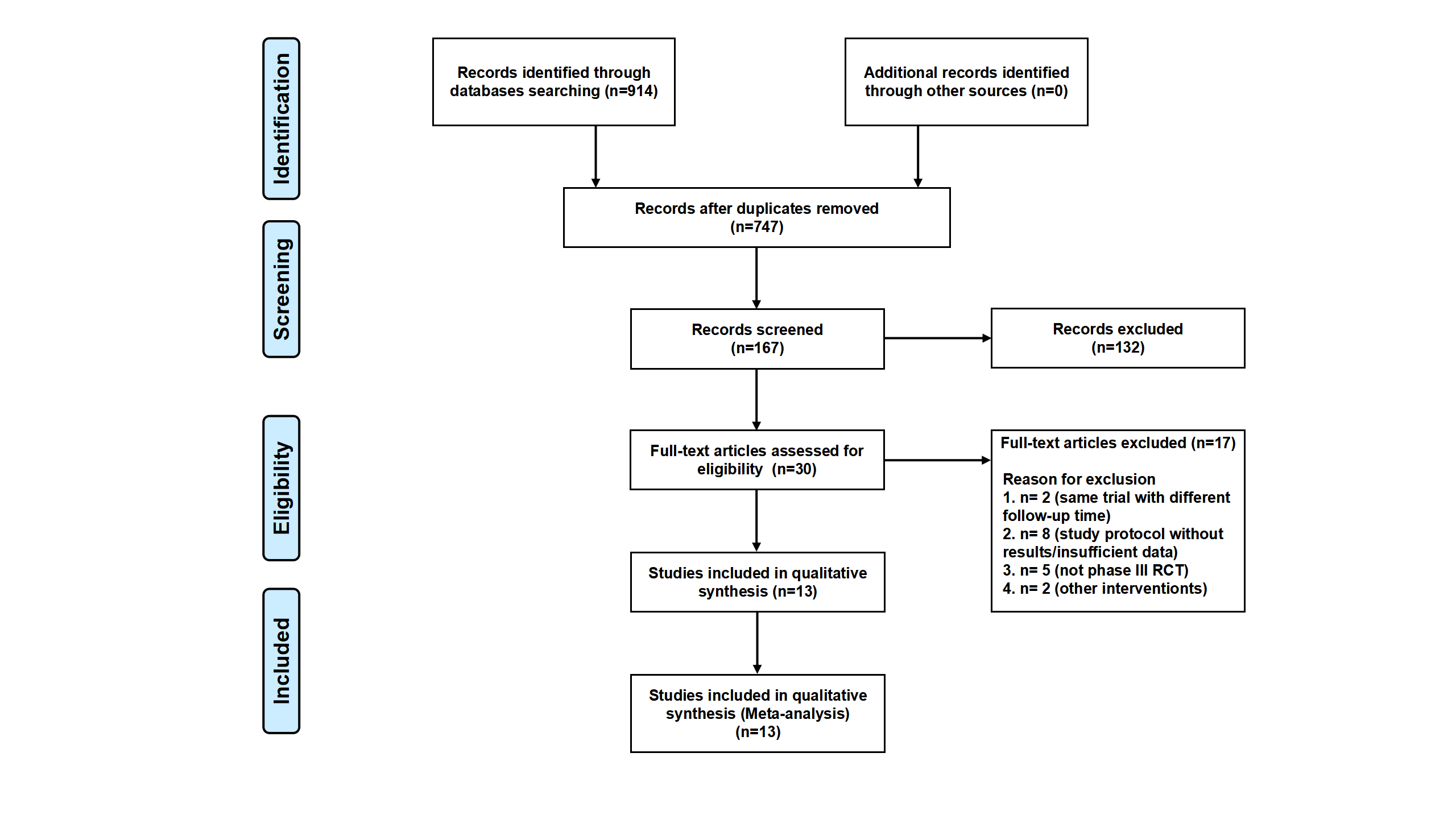

Supplement: Supplementary Figure 1 — Systematic literature process of the search. [file Image_1.tif]
